# Solution structures of the DNA-binding domains of immune-related zinc-finger protein ZFAT

**DOI:** 10.1007/s10969-015-9196-3

**Published:** 2015-03-24

**Authors:** Naoya Tochio, Takashi Umehara, Kazuhiko Nakabayashi, Misao Yoneyama, Kengo Tsuda, Mikako Shirouzu, Seizo Koshiba, Satoru Watanabe, Takanori Kigawa, Takehiko Sasazuki, Senji Shirasawa, Shigeyuki Yokoyama

**Affiliations:** 1 RIKEN Systems and Structural Biology Center, 1-7-22 Suehiro-cho, Tsurumi, Yokohama, 230-0045 Japan; 2 RIKEN Center for Life Science Technologies, 1-7-22 Suehiro-cho, Tsurumi, Yokohama, 230-0045 Japan; 3 PRESTO, Japan Science and Technology Agency (JST), 1-7-22 Suehiro-cho, Tsurumi, Yokohama, 230-0045 Japan; 4 Department of Maternal-Fetal Biology, National Research Institute for Child Health and Development, Tokyo, 157-8535 Japan; 5 Department of Computational Intelligence and Systems Science, Interdisciplinary Graduate School of Science and Engineering, Tokyo Institute of Technology, 4259 Nagatsuta-cho, Midori, Yokohama, 226-8502 Japan; 6 Institute for Advanced Studies, Kyushu University, 6-10-1 Hakozaki, Higashi-ku, Fukuoka, 812-8582 Japan; 7 Department of Cell Biology, Faculty of Medicine, Fukuoka University, Fukuoka, 814-0180 Japan; 8 Center for Advanced Molecular Medicine, Fukuoka University, Fukuoka, 814-0180 Japan; 9 RIKEN Structural Biology Laboratory, 1-7-22 Suehiro-cho, Tsurumi, Yokohama, 230-0045 Japan; 10 Present Address: Department of Mathematical and Life Sciences, Research Center for the Mathematics on Chromatin Live Dynamics, Graduate School of Science, Hiroshima University, 1-3-1 Kagamiyama, Higashi-Hiroshima, 739-8530 Japan; 11 Present Address: Department of Integrative Genomics, Tohoku Medical Megabank Organization, Tohoku University, 2-1 Seiryo-machi, Aoba-ku, Sendai, 980-8573 Japan; 12 Present Address: RIKEN Quantitative Biology Center, 1-7-22 Suehiro-cho, Tsurumi, Yokohama, 230-0045 Japan

**Keywords:** Gene expression, NMR, Transcription, Zinc-finger

## Abstract

ZFAT is a transcriptional regulator, containing eighteen C_2_H_2_-type zinc-fingers and one AT-hook, involved in autoimmune thyroid disease, apoptosis, and immune-related cell survival. We determined the solution structures of the thirteen individual ZFAT zinc-fingers (ZF) and the tandemly arrayed zinc-fingers in the regions from ZF2 to ZF5, by NMR spectroscopy. ZFAT has eight uncommon bulged-out helix-containing zinc-fingers, and six of their structures (ZF4, ZF5, ZF6, ZF10, ZF11, and ZF13) were determined. The distribution patterns of the putative DNA-binding surface residues are different among the ZFAT zinc-fingers, suggesting the distinct DNA sequence preferences of the N-terminal and C-terminal zinc-fingers. Since ZFAT has three to five consecutive tandem zinc-fingers, which may cooperatively function as a unit, we also determined two tandemly arrayed zinc-finger structures, between ZF2 to ZF4 and ZF3 to ZF5. Our NMR spectroscopic analysis detected the interaction between ZF4 and ZF5, which are connected by an uncommon linker sequence, KKIK. The ZF4–ZF5 linker restrained the relative structural space between the two zinc-fingers in solution, unlike the other linker regions with determined structures, suggesting the involvement of the ZF4–ZF5 interfinger linker in the regulation of ZFAT function.

**Electronic supplementary material:**

The online version of this article (doi:10.1007/s10969-015-9196-3) contains supplementary material, which is available to authorized users.

## Introduction

**Fig. 1:**
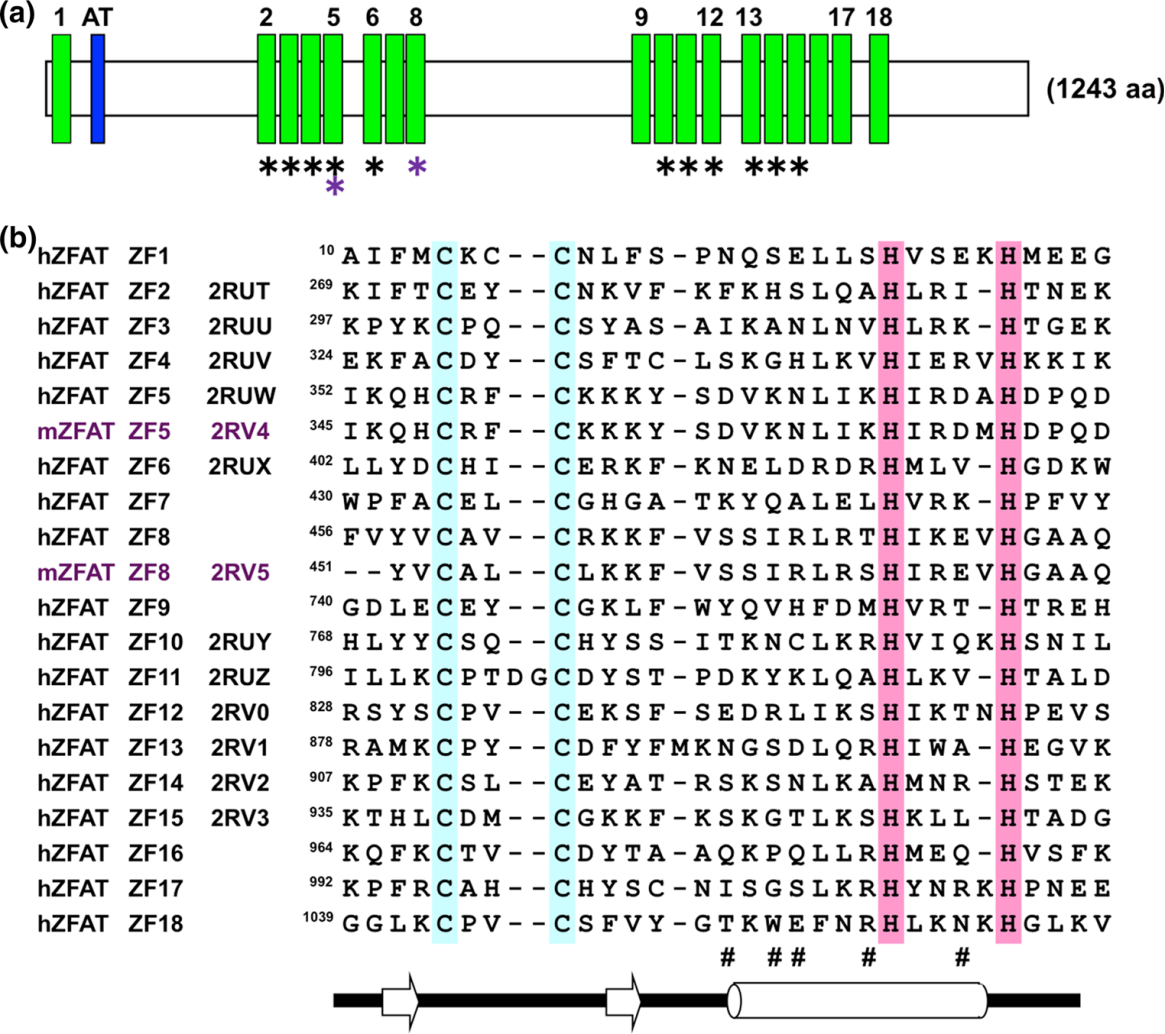
Primary structure of ZFAT. **a** Schematic representation of human ZFAT. The *green* and *blue boxes* indicate the C_2_H_2_ zinc-finger and the AT-hook motif, respectively. The positions of the zinc-fingers with solved structures are marked by *asterisks* (*black* human; *violet* mouse). **b** Sequence alignment of the ZFAT zinc-fingers. All of the human ZFAT zinc-fingers and mouse ZFAT zinc-fingers (mZF5 and mZF8 in *violet*) with solved structures are listed, with h and m indicating human and mouse, respectively. The zinc-coordinating Cys and His residues are colored cyan and magenta, respectively. The *hash mark* indicates the residues expected to be involved in DNA recognition. Secondary structures corresponding to the sequence are shown at the *bottom*

Autoimmune thyroid disease (AITD) is a general disease caused by the immune system responding to its own normal cells or organs, and to foreign antigens such as bacteria, viruses, and tumors [1–3]. The development of antibodies to antigenic thyroid components is a main feature of autoimmune diseases. *ZFAT* (Zinc finger gene in AITD susceptibility region; also known as ZNF406) was identified as a gene involved in the regulation of the autoimmune system [4]. The ZFAT protein is conserved from fish to human, and the human ZFAT protein is composed of eighteen C_2_H_2_-type zinc-fingers (ZFs) and one AT-hook motif between ZF1 and ZF2 [5] (Fig. 1a). ZFAT is expressed in peripheral B and T lymphocytes, and is also found in the human acute T lymphoblastic leukaemia cell line MOLT-4 and human umbilical vein endothelial cells [6, 7]. Notably, the ZFAT-knockdown in MOLT-4 induces apoptosis via the activation of caspases, suggesting that ZFAT is a transcriptional regulator involved in apoptosis and cell survival for immune-related cells. [6]. Furthermore, *ZFAT* is an essential transcriptional regulator for hematopoietic differentiation and indispensable for mouse embryonic development [8, 9], which indicates the critical role of ZFAT not only in AITD but also in a broad range of development and differentiation.

The transcriptional activity of ZFAT is considered to be mediated by its DNA-binding ZFs. The C_2_H_2_-type ZF, consisting of 20–30 residues, forms one N-terminal short antiparallel β-sheet and one helix [10]. The canonical C_2_H_2_ ZFs bind to specific DNA sequences, and the amino acids located at positions −1, +2, +3 and +6, from the N-terminal residue of the helix, directly contact a DNA base. The DNA recognition modes by these base-contacting residues were predicted from previous structural analyses, and the relationships between the base-contacting residues and the predicted DNA bases have been summarized as the recognition code [11]. In most cases, the C_2_H_2_-type ZF is repeated from two to more than thirty times in a protein [10]. Such tandem sets of ZFs are typically connected by a well-conserved TGEKP linker sequence [11–15]. These consecutive ZFs are known to bind to cognate DNA sequences as one functional unit [10, 16–19]. To understand the functional role of ZFAT in the regulation of the immune system, we determined the solution structures of single or consecutive tandem ZFs of ZFAT through an NMR method. We describe the structural features of the ZFAT ZFs, including the structural differences on the putative DNA recognition surfaces among the ZFAT ZFs, and the unique interaction mode within the tandem ZFs of ZF4 and ZF5, which are connected by an uncommon linker sequence.

## Materials and methods

### Protein expression and purification

The DNA sequences encoding the ZFs of the human and mouse ZFAT proteins (SwissProt accession numbers: Q9P243 and Q7TS63) were subcloned by PCR from the human and mouse cDNA clones by the two-step PCR method [20]. The individual domain regions used in this study are listed in Table 1. The cDNA fragments encoding these regions, along with those containing tandem ZF sequences, were cloned into the expression vector pCR2.1-TOPO (Invitrogen, Carlsbad, CA), as a fusion with an N-terminal poly-histidine affinity tag and a tobacco etch virus (TEV) protease cleavage site, and an artificial linker sequence (GSSGSSG) [20]. The actual sequences of the NMR samples contain these seven extra residues at their N-termini. The ^13^C/^15^N-labeled fusion proteins were synthesized by the cell-free protein expression system [21, 22], and were purified using a chelating column, as described previously [23, 24]. The purified proteins were concentrated to 0.1–1.2 mM in 20 mM Tris-*d*_11_–HCl buffer (pH 7.0), containing 100 mM NaCl, 1 mM dithiothreitol-*d*_10_, 50 μM ZnCl_2_, 1 mM iminodiacetic acid (IDA), 10 % D_2_O, and 0.02 % NaN_3_.

**Table 1:** Summary of the deposited data of the ZFAT zinc-finger structures

Domain name	Residues	PDB (calculated by CYANA)	PDB (refined by AMBER)	BMRB
h ZFAT ZF2	269–297	2ELS	2RUT	11474
h ZFAT ZF3	297–325	2ELT	2RUU	11475
h ZFAT ZF4	324–353	2RSH	2RUV	11486
h ZFAT ZF5	352–381	2ELU	2RUW	11476
h ZFAT ZF6	402–430	2ELV	2RUX	11477
h ZFAT ZF10	768–797	2ELM	2RUY	11478
h ZFAT ZF11	796–826	2ELN	2RUZ	11479
h ZFAT ZF12	828–857	2ELO	2RV0	11480
h ZFAT ZF13	878–907	2ELP	2RV1	11481
h ZFAT ZF14	907–935	2ELQ	2RV2	11482
h ZFAT ZF15	932–963	2ELR	2RV3	11483
m ZFAT ZF5	352–381	2ELW	2RV4	11484
m ZFAT ZF8	458–485	2ELX	2RV5	11485
h ZFAT ZF2–ZF3–ZF4	269–353	2RSJ	2RV6	11488
h ZFAT ZF3–ZF4–ZF5	297–381	2RSI	2RV7	11487

*h* human, *m* mouse

### NMR spectroscopy and spectral assignments

All spectra were recorded on Bruker Avance 600, 700, 800, and 900 spectrometers at 296 or 298 K. Samples were first screened by ^1^H, ^15^N-HSQC spectroscopy [25]. The resonance assignments were accomplished using a conventional set of triple resonance spectra, as described previously [23, 24], and have been deposited in the Biological Magnetic Resonance data Bank (BMRB; Table 1). Inter-proton distance restraints were obtained from ^15^N and ^13^C edited NOESY spectra, both recorded with a mixing time of 80 ms. All spectra were processed using NMRPipe [26], and the programs Kujira [27] and NMRView [28] were employed for optimal visualization and spectral analyses.

### Structure calculations

Automated NOE cross-peak assignments and structure calculations with torsion angle dynamics were performed using the software package CYANA [29, 30]. The backbone dihedral angle restraints from the TALOS program [31] were also included for the calculations, with allowed ranges of ±30°. The final structure calculations with CYANA were started from 100 conformers with random torsion angle values. The 20 conformers with the lowest final CYANA target function values were further refined with the AMBER12 program, using an Amber ff99SB force field and a generalized Born model, as described previously [32]. The tetrahedral zinc coordination was restrained by lower and upper distance limits, with force constants of 1000 kcal/mol/Å. All of the structures were validated using MolProbity [33, 34] and PROCHECK-NMR [35]. The structural statistics of the ZFAT ZFs are summarized in Supplemental Tables 1–3. Figures were generated with the MOLMOL [36] and PyMol (DeLano Scientific, San Carlos, CA) programs. All structures have been deposited in the Protein Data Bank. The PDB and BMRB accession codes of the structure-determined ZFAT ZF structures are provided in Table 1.

## Results and discussion

### Structural overview of the ZFAT zinc-fingers

**Fig. 2:**
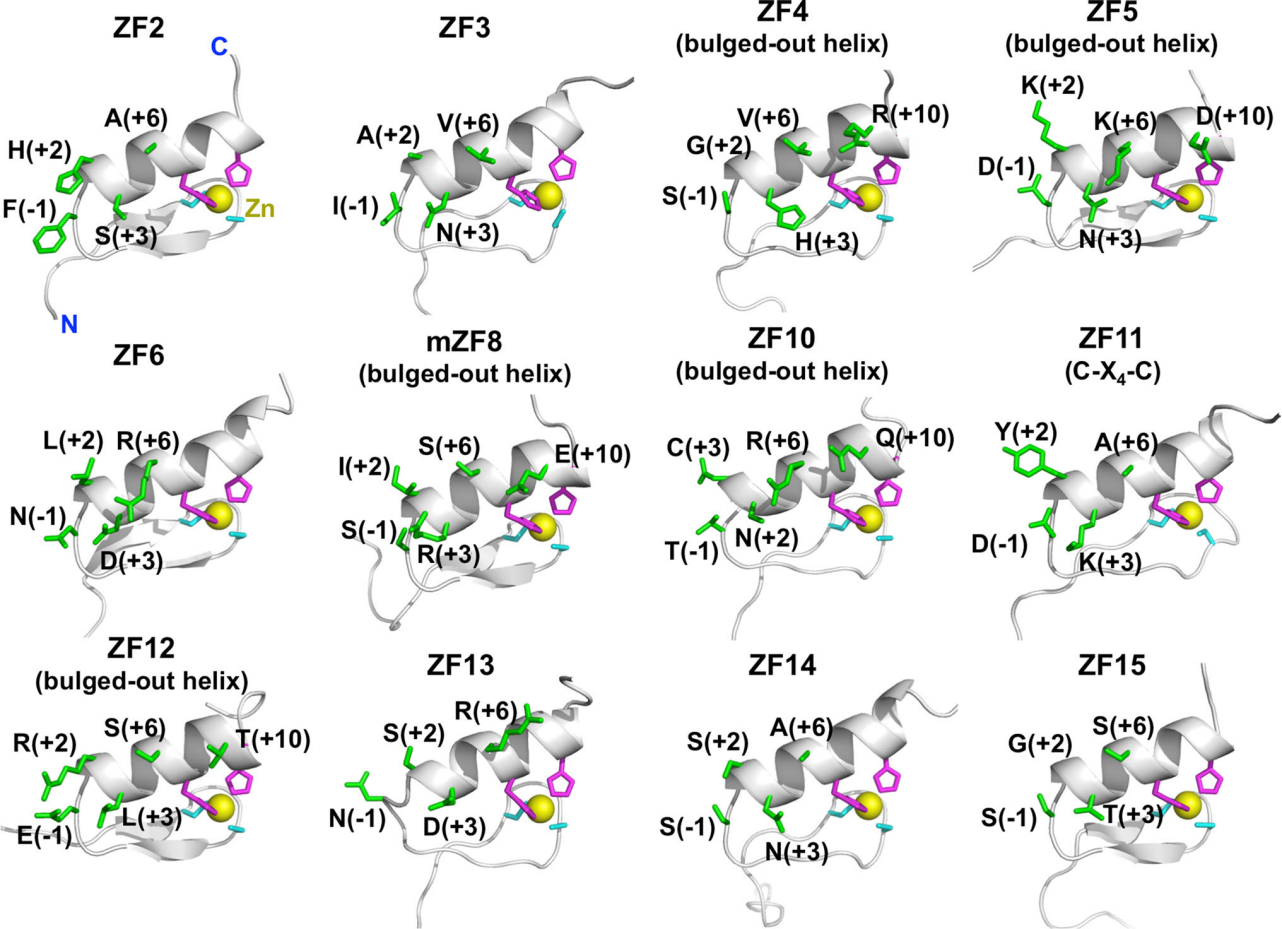
Solution structures of isolated ZFAT zinc-fingers, depicted by ribbon diagrams. The zinc ion and the zinc-coordinating histidines and cysteines in each zinc-finger structure are colored *yellow*, *magenta* and *cyan*, respectively. The residues located at the positions that are potentially involved in DNA recognition are shown in *green*. The orientations of all of the structures are the same

**Fig. 3:**
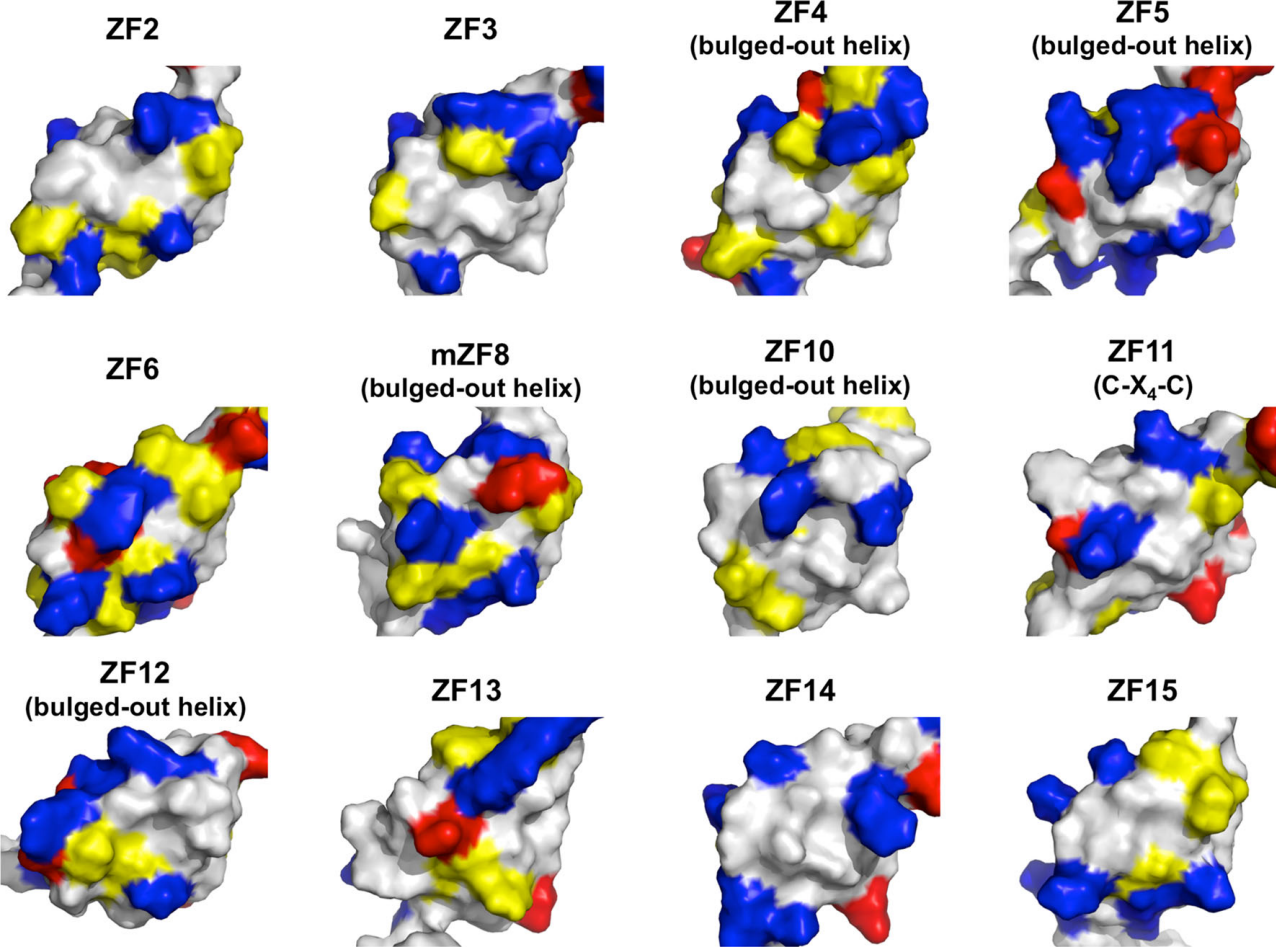
Surface representation of isolated ZFAT zinc-fingers. Basic, acidic, and hydrophobic residues are colored *blue*, *red*, and *yellow*, respectively. The orientation of all of the structures is the same as in Fig. 2

The domain architecture of ZFAT is shown in Fig. 1a. The ^1^H, ^15^N and ^13^C assignments of each individual ZFAT ZF (Fig. 1b) expressed in the cell-free system were obtained by combining selected triple-resonance spectra. By screening the nature of the candidate protein samples, such as expression, solubility, and folding, we finally determined the following thirteen ZFAT ZF solution structures: human ZF2, ZF3, ZF4, ZF5, ZF6, ZF10, ZF11, ZF12, ZF13, ZF14 and ZF15; and mouse ZF5 and ZF8. All of the individual ZFs consisted of one N-terminal short antiparallel β-sheet and one helix (Figs. 1b, 2; Table 1), and their overall structures were similar to each other. On the other hand, the compositions of the putative DNA base-contacting surfaces differed among the solved zinc-finger structures (Fig. 3), suggesting functional divergence regarding their involvement and sequence specificity in DNA recognition.

**Fig. 4:**
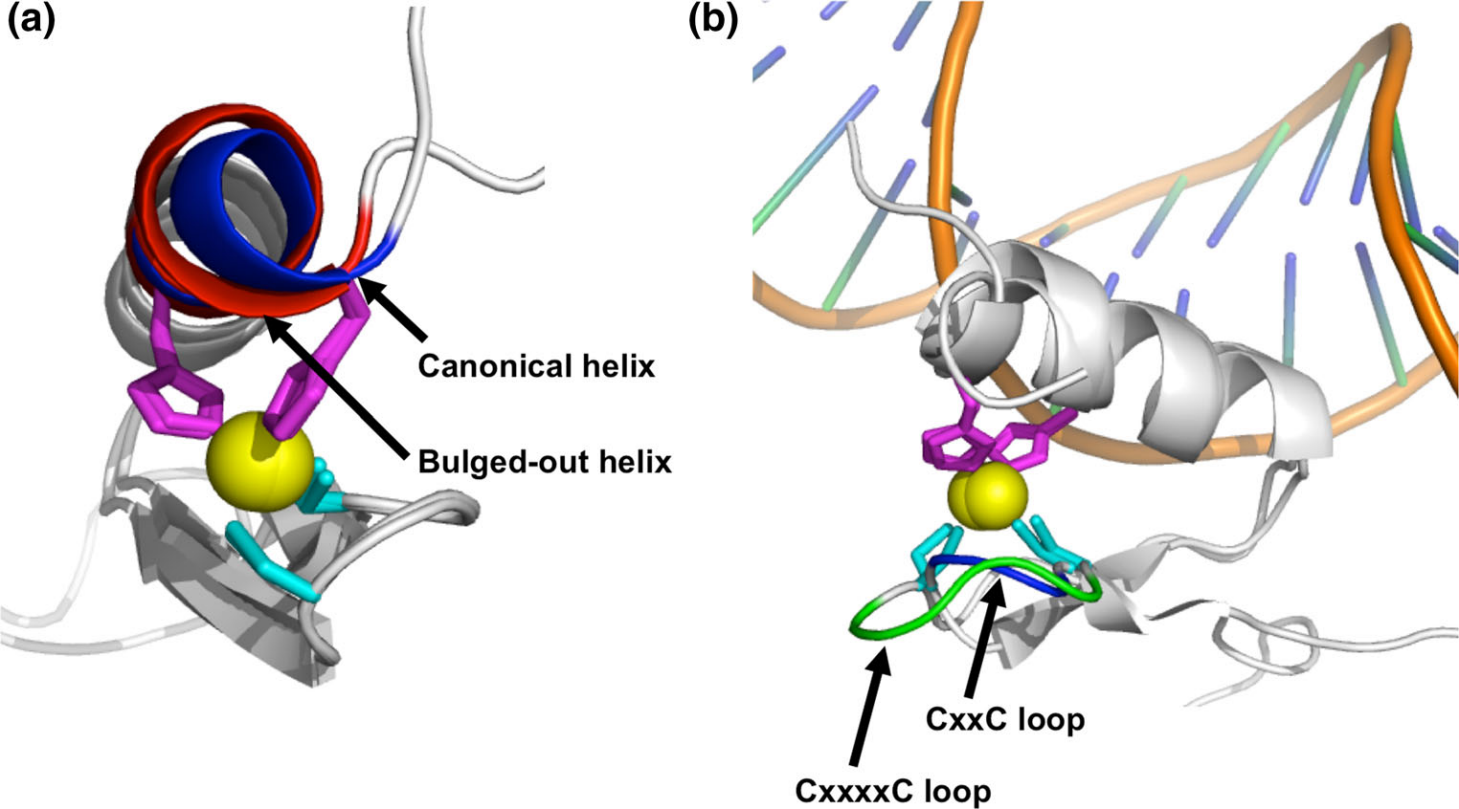
Structural features of ZFAT zinc-fingers. **a** Comparison of the bulged-out helix structure (hZF5, *red*) with that of the canonical helix (hZF2, *blue*). The zinc atom is depicted by a *yellow ball*. The zinc-coordinating Cys and His residues are depicted by *cyan* and *magenta sticks*, respectively. **b** Comparison of the loop structure of the C–X_4_–C type (hZF11, *green*) with that of the standard C–X_2_–C type zinc-finger (hZF12, *blue*). *Other color codes* are the same as in (**a**). The Cα positions of the protein–DNA complex structure of the DNA-binding zinc-finger of GLI (PDB ID: 2GLI) are used as the reference Cα positions. The position of the DNA (*orange*) is also from the GLI structure, for reference

Among the determined ZFAT ZF structures, six ZFs (i.e. hZF4, hZF5, mZF5, mZF8, hZF10, and hZF12) have a bulged-out helix structure, instead of a canonical helix structure (Fig. 4a). In a canonical helix, the zinc atom is held in a tetrahedral complex by the two Sγ of the C–X_2–4_–C sequence and the two Nε2 of the H–X_3_–H sequence, where C represents Cys, H is His, and X is any amino acid residue, and the subscript number represents the number of amino acid residues (Figs. 1b, 4a). On the other hand, the zinc atom in a bulged-out helix is held by the two Sγ of the C–X_2_–C sequence and the two Nε2 of the H–X_4_–H sequence (Figs. 1b, 4a). The bulged-out helix is amphipathic, with the side-chains of their hydrophobic faces packing the core of the domain and the exposed surface of the helix facing the hydrophilic residues involved in DNA recognition [37]. These structural features of the canonical or bulged-out helices are common among all of the ZFAT ZFs (Fig. 1b), and are also similar to those of other canonical C_2_H_2_ ZFs.

### C–X_4_–C type zinc-finger and bulged-out helix-containing zinc-fingers

There are two interesting structural features in the folds of the ZFAT ZFs. The first is that all of the ZFAT ZFs, except ZF11, have a short two-residue-spacer between the two zinc-coordinating cysteines, which is typically observed in Krüppel-type ZFs (i.e. C–X_2_–C). On the other hand, the ZF11 ZF has a long four-residue-spacer in the corresponding region (i.e. C–X_4_–C; Fig. 1b), yielding an extended β loop structure between the two antiparallel β strands (Fig. 4b). The N-terminal antiparallel β loop structure, which is formed by the interaction of zinc with the two zinc-coordinating cysteines, is essential for the stability of the overall ZF structure. When the zinc ion binds to an unfolded apo-form finger, it first interacts with the Cys residues and subsequently with the His residues [10]. The difference in the length between the two Cys residues is assumed to either modulate the stability or facilitate the interactions with other intramolecular ZFs [38]. The β loop structure may also function as a scaffold and affect the DNA-binding activity [39].

The second feature is that the ZFAT ZFs have an abundance of the abovementioned bulged-out helix structures (Figs. 1b, 4a). In the SMART database, (containing 274,117 C_2_H_2_ ZFs), approximately 80 % of the C_2_H_2_ ZFs (212,646) have the canonical H–X_3_–H motif, while only 15 % of the ZFs (40,767) have the H–X_4_–H motif. Notably, the ZFAT protein has eight bulged-out helix ZFs (44 %), including five with determined structures (i.e. hZF4, h/mZF5, mZF8, hZF10, and hZF12) and three putative bulged-out helix-containing ZFs (i.e. ZF1, ZF17, and ZF18), as judged from its amino acid sequence (Figs. 1b, 2). The percentage of bulged-out helix-containing ZFs of ZFAT, 44 %, is higher than that of frog TFIIIA (30 %), another known bulged-out helix ZF-containing protein [40]. The four-residue-spacing between the two histidines of the bulged-out helix, in which one amino acid is inserted into the canonical helix, is assumed to be critical for the structure and function of ZFAT. In order to maintain an ideal position for zinc coordination (see the two histidines in Fig. 4a), the additionally inserted residue causes the helix to bulge out slightly relative to those of the canonical helix ZFs. Consequently, this H–X_4_–H region forms a slightly larger and looser helical structure, as compared with the canonical H–X_3_–H helix, without distorting the overall ZF structure (Fig. 4a).

Although the backbone *i*–*i*-5 hydrogen bond, known as a π hydrogen bond, was formed between two His residues in each bulged-out helix, the backbone dihedral angles were quite different from those of the ideal π-helix (φ = −57.1; ψ = −69.7) [41, 42], as well as those of the ideal α helix (φ = −65.0; ψ = −40.0) [43, 44] (e.g., φ = −111.0 ± 16.1 for His349; and ψ = −36.6 ± 12.7 for Val348, respectively, in hZF4; see also Table 2). Therefore, as defined in the structural study of TFIIIA by Wuttke et al. [40], we used the term ‘bulged-out helix’ to describe an H–X_4_–H ZFAT ZF helix in this study, rather than the term ‘π-helix’. The unique bulged-out helix structure can allow distinct non-coordinating amino acids located in invariant positions to form hydrogen bonds with specific nucleotide bases in the major groove of DNA [10]. It also allows the canonical ZF helix, which is located adjacent to the bulged-out helix, to form extensive interactions with DNA [40].

**Table 2:** φ and ψ angles of the bulged-out helices of ZFAT ZFs

	φ of the second His residue in C_2_H_2_	ψ of the residue just before the His residue
h ZFAT ZF4	−111.0 ± 16.1	−36.6 ± 12.7
h ZFAT ZF5	−132.3 ± 1.9	−25.5 ± 1.3
h ZFAT ZF10	−129.6 ± 4.4	−18.4 ± 4.7
h ZFAT ZF12	−134.0 ± 1.9	−27.5 ± 1.5
m ZFAT ZF5	−132.6 ± 3.9	−23.4 ± 3.1
m ZFAT ZF8	−105.3 ± 5.6	−31.3 ± 3.5
Ideal π helix^a^	−57.1	−69.7
Ideal α helix^b^	−65.0	−40.0

*h* human, *m* mouse

^a^References [41] and [42]

^b^References [43] and [44]

### Expected DNA recognition sequences of the ZFAT zinc-fingers

**Fig. 5:**
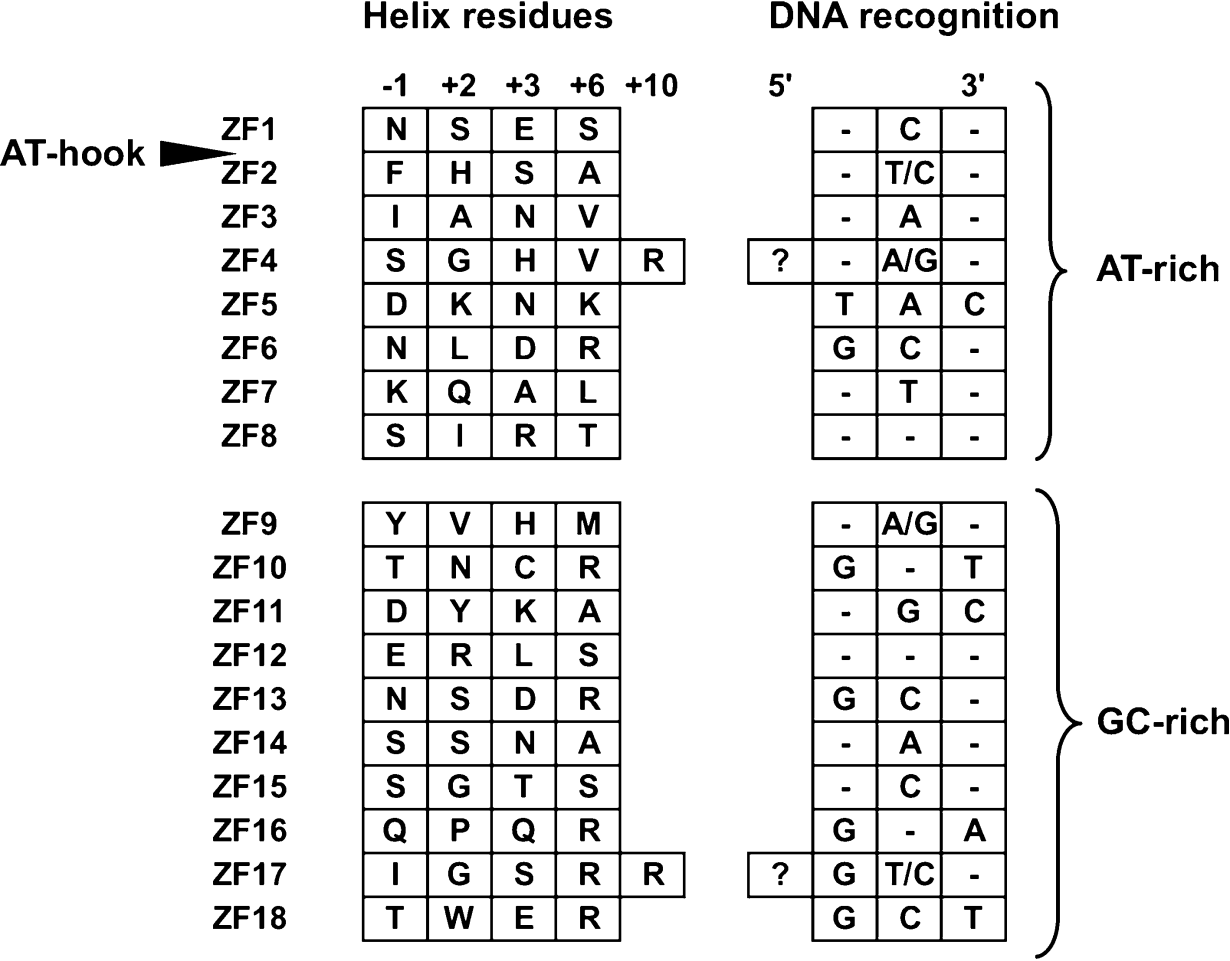
Expected DNA recognition sequences for each of the ZFAT zinc-fingers. The amino acid residues located at the key positions in the helix (−1, +2, +3, +6 and +10) of each zinc-finger are listed on the *left*, and the three to four base sequence (subsite) predicted to be preferred by each zinc-finger is shown on the *right*

As for the molecular surfaces of the ZFAT ZFs, although all of the folds of the ZFAT ZFs are well conserved, the exposed surface of the helix for putative DNA-binding has a wide variety of physiochemical properties in the individual ZFAT ZFs (Fig. 3; see also Table 1). This suggested that some of the ZFAT ZFs contribute to the recognition of different DNA sequences or protein interactions [19]. In order to predict the DNA sequences recognized by the ZFAT ZFs, we applied the DNA-ZF recognition code [11], using our structural data. The possible DNA-contacting residues of the ZFAT ZFs at the key positions within the canonical or bulged-out helices (left panels), and the nucleotide preferred by each key residue of each ZF (right panels), are shown in Fig. 5. As for the bulged-out helix-containing ZFs, the residues involved in the extended interaction with DNA [40] are also shown in Fig. 5 (see the +10 residues in ZF4 and ZF17). From this prediction, it is plausible that the N-terminal half of the ZFAT ZFs may prefer DNA subsites containing AT-rich sequences (Fig. 5). This assumption is consistent with the fact that the AT-hook region prefers to bind an AT sequence existing between ZF1 and ZF2 (Fig. 1a). On the other hand, the C-terminal half of the ZFAT ZFs may prefer the DNA subsites containing GC-rich sequences (Fig. 5). Since consecutive ZFs bind to their corresponding DNA sequences in an anti-parallel fashion, where one ZF binds to one triplet DNA sequence and the adjacent C-terminal ZF binds to another triplet on the 5′-side [45, 46], the DNA sequence preferentially recognized by ZFAT may be a GC-rich sequence followed by an AT-rich sequence. Many C_2_H_2_ ZF proteins contain tandemly arrayed ZFs connected by specific linker sequences, while other members contain single or duplicated pairs of ZFs [10, 17]. Since the C_2_H_2_ ZFs are frequently involved in DNA-binding, variations in the numbers of ZFs and their spacing may affect DNA recognition [47]. Especially, multiple tandemly arrayed C_2_H_2_ ZFs can bind to the cognate DNA through two to three consecutive fingers [10, 16–19]. Based on the ZFAT domain architecture and the amino acid lengths of the linkers between the individual ZFAT ZFs, the following four ZFs are assumed to collaborate as DNA recognition units: [ZF2–ZF5] and [ZF6–ZF8] in the N-terminal half, and [ZF9–ZF12] and [ZF13–ZF17] in the C-terminal half (Fig. 1a). However, the precise target DNA sequence of ZFAT could not be identified, because of the lack of information about how these tandem ZF units cooperate with each other in recognizing a particular DNA sequence and how the bulged-out helix recognizes bases in a particular DNA sequence.

### Structural analysis of tandemly arrayed ZFAT zinc-fingers

**Fig. 6:**
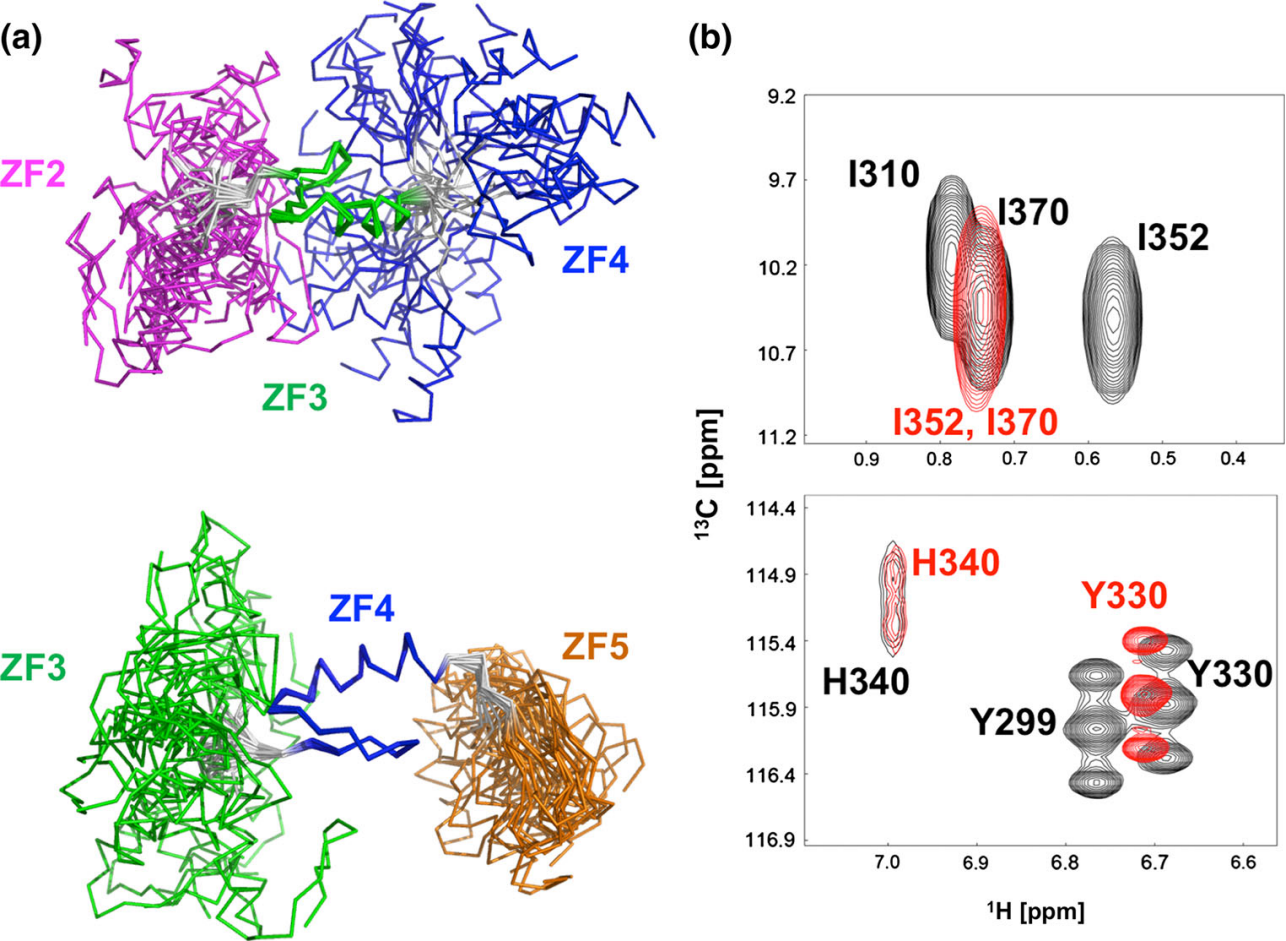
The uncommon interfinger linker reduces the flexibility. **a** The solution structures of the tandem ZF regions, ZF2–ZF3–ZF4 (*top*) and ZF3–ZF4–ZF5 (*bottom*). ZF2, ZF3, ZF4, and ZF5 are colored *magenta*, *green*, *blue*, and *orange*, respectively. In each structure, the central ZF is used for fitting. **b** Comparison of the ^1^H,^13^C HSQC spectra between ZF3–ZF4–ZF5 (*top*, *black*) and ZF5 (*top*, *red*), and ZF3–ZF4–ZF5 (*bottom*, *black*) and ZF4 (*bottom*, *red*). Signal assignments are labeled in the spectra

**Fig. 7:**
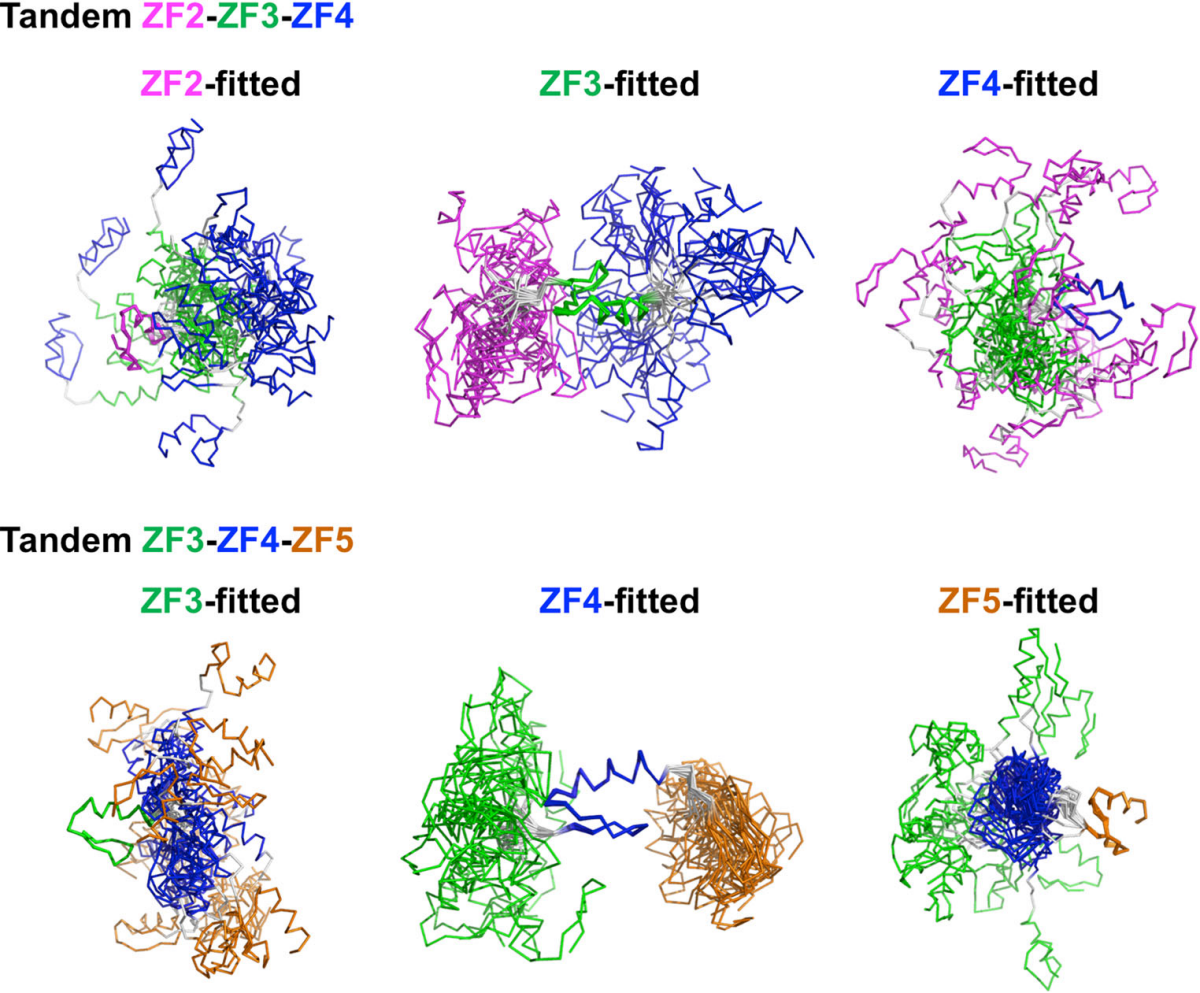
Comparison of interfinger flexibilities. The solution structures of the tandem ZF regions, ZF2–ZF3–ZF4 (*top*) and ZF3–ZF4–ZF5 (*bottom*), are shown. In each structure, the first, central, and last ZF-fitted superimposed structures are shown on the *left*, *middle*, and *right*, respectively. The *color code* is the same as in Fig. 6a

**Fig. 8:**
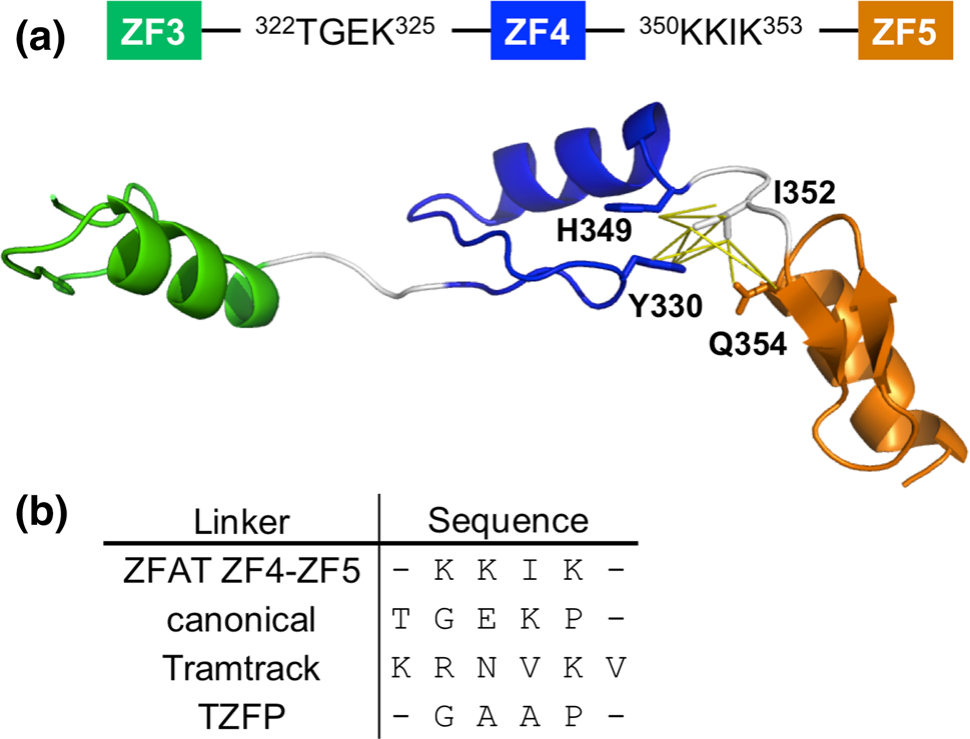
The role of the uncommon interfinger linker. **a** The solution structure of the tandem ZF3–ZF4–ZF5 region. ZF3, ZF4, and ZF5 are colored *green*, *blue*, and *orange*, respectively. The side chains for which inter-residue NOEs were observed are depicted by *sticks*. The observed NOEs are shown in *yellow lines*. **b** Comparison of the sequences of the interfinger linkers. The sequences of the interfinger linkers of ZFAT (ZF4–ZF5), Tramtrack, and a canonical C_2_H_2_ zinc-finger are listed

In order to reveal the structural features of the tandemly arrayed ZFAT ZFs, we tried to determine the tertiary structures of tandem ZFAT ZF regions. We determined the solution structures of the tandem repeats ZF2–ZF3–ZF4 and ZF3–ZF4–ZF5 (Figs. 6a, 7; Table 1). The structures of the individual ZFs in the tandem ZF regions are quite similar to the corresponding isolated ZFs. Furthermore, the chemical shifts of almost all of the signals in both the tandem ZF regions and the isolated ZFs did not change, except for those detected in the terminal regions (data not shown). However, we found that the chemical shifts of the Ile352 (in the interfinger linker connecting ZF4 and ZF5) and Tyr330 (in ZF4) residues were quite different between the cases of the isolated ZF and the tandem ZF (Fig. 6b). Additionally, we observed several NOEs from Ile352 (in the interfinger linker connecting ZF4 and ZF5) to Tyr330 (in the β loop of ZF4), His349 (in the helix of ZF4), and Gln354 (in the β strand of ZF5). Although we could not determine the position of ZF5 relative to ZF4, because of the lack of clear interfinger NOEs between ZF4 and ZF5, these NOEs suggested that Ile352 may function as a clamp to limit the interdomain mobility between ZF4 and ZF5 (Figs. 6a, 7, 8a).

In contrast to the case with ZF4-ZF5, there were no such NOEs detected in ZF3–ZF4 and ZF2–ZF3. As a result, when the central ZF4 was fitted in the tandem ZF3–ZF4–ZF5, the RMSD and its standard deviation of ZF5 were both smaller than those of ZF3 (i.e. RMSD for ZF4-fitted ZF5 is 10.10 ± 4.82 Å, whereas that for ZF4-fitted ZF3 is 21.82 ± 9.62 Å; Figs. 6a, 7). Similarly, these values of the ZF4-fitted ZF5 in the tandem ZF3–ZF4–ZF5 were also smaller than those of both ZF3-fitted ZF2 and ZF3-fitted ZF4 in the tandem ZF2–ZF3–ZF4 (i.e. RMSDs for ZF3-fitted ZF2 = 15.25 ± 6.23 Å and ZF3-fitted ZF4 = 24.11 ± 9.01 Å; Figs. 6a, 7). Interestingly, previous linker mutation and NMR relaxation experiments revealed that the GAAP linker sequence of mouse testis zinc finger protein (TZFP) is more rigid than the canonical TGEKP linker sequence, lacking interfinger NOEs, in the absence of DNA [48]. Therefore, the linker sequence itself may affect the relative interfinger flexibility, without exhibiting interfinger NOEs.

The linker sequences between the canonical DNA-binding C_2_H_2_ ZFs are highly conserved, and are typically TGEKP. This sequence is necessary for DNA-binding and the interactions between two neighboring ZFs [11–15]. This canonical TGEKP linker is flexible in solution in the absence of DNA, whereas the linker in the DNA-bound complex forms a compact structure with a “snap-lock” helix-cap for stabilization of the DNA complex structure [13]. In addition, the TGEKP linker can be phosphorylated or acetylated, to regulate the DNA-binding activity of the tandemly arrayed C_2_H_2_ ZFs [49–51]. Intriguingly, this canonical linker sequence is not conserved in the several ZFAT interfinger regions. The linker sequence intervening between ZF4 and ZF5 is KKIK, which is completely different from the canonical linker sequence (Fig. 8b). In the case of the two tandem ZFs in Tramtrack, in which the linker sequence is KRNVKV (Fig. 8b), this linker is more flexible than the canonical TGEKP linker sequence, even upon DNA binding [52]. This flexibility reflected the absence of the helix-cap by the interfinger linker upon DNA binding and might contribute to the DNA binding mode where the DNA structure was distorted from the B form [13, 52].

In contrast, the structure of the KKIK linker between ZF4 and ZF5, which is another atypical linker sequence, was slightly restrained, even in the absence of DNA (Figs. 6a, 8). In the case of three tandemly repeats of TZFP which has the more rigid linker between ZF2 and ZF3, the mutation of the native GAAP linker to the canonical TGEKP linker obviously decreased the DNA binding of ZF3 [48]. The other interfinger linker sequences of ZFAT also differ from the highly conserved canonical TGEKP linker sequence, which may be related to the functions of the ZFAT ZFs in gene regulation. Further structural and biochemical analyses involving DNA-bound forms of ZFAT with tandem ZFAT ZFs, bulged-out helix-containing ZFs, and ZFAT interfinger linker sequences will be necessary to understand the molecular function of ZFAT.

## Electronic supplementary material

Below is the link to the electronic supplementary material.

Supplementary material 1 (DOC 110 kb)

## Abbreviations

AITD: Autoimmune thyroid disease
ZFAT: Zinc-finger gene in AITD susceptibility region
ZF: Zinc-finger
TEV: Tobacco etch virus
NMR: Nuclear magnetic resonance
HSQC: Hetero-nuclear single quantum coherence
NOE: Nuclear Overhauser effect
NOESY: NOE spectroscopy
PDB: Protein Data Bank
BMRB: Biological Magnetic Resonance data Bank
