# Supplementary material for: Solution structures of the DNA-binding domains of immune-related zinc-finger protein ZFAT
Source: J Struct Funct Genomics. 2015 Mar 24;16(2):55–65. doi: 10.1007/s10969-015-9196-3 (PMC4427657; doi:10.1007/s10969-015-9196-3)
Supplement: Supplementary file 1 — Supplementary material 1 (DOC 110 kb) [file 10969_2015_9196_MOESM1_ESM.doc]

| **Supplemental Table 1. Summary of statistics of the final 20 structures of ZFAT ZFs** | | | | | | | |
| --- | --- | --- | --- | --- | --- | --- | --- |
| Domain Names | hZFAT ZF2 | hZFAT ZF3 | hZFAT ZF4 | hZFAT ZF5 | hZFAT ZF6 | hZFAT ZF10 | hZFAT ZF11 |
| PDB IDs | 2RUT | 2RUU | 2RUV | 2RUW | 2RUX | 2RUY | 2RUZ |
| NOE upper distance restraints |  |  |  |  |  |  |  |
| Intra-residual (|*i*-*j*| = 0) | 159 | 143 | 80 | 167 | 189 | 107 | 167 |
| Medium-range (1 ≤ |*i*-*j*| ≤ 4) | 326 | 327 | 100 | 278 | 354 | 131 | 280 |
| Long-range (|*i*-*j*| > 4) | 155 | 181 | 20 | 112 | 182 | 59 | 159 |
| Total | 640 | 651 | 200 | 557 | 725 | 297 | 606 |
| Dihedral angle restraints (ϕ and ψ) | 28 | 28 | 30 | 24 | 22 | 28 | 28 |
|  |  |  |  |  |  |  |  |
| CYANA target function value (Å^2^) | 0.0004 ± 0.0006 | 0.0285 ± 0.0050 | 0.0000 ± 0.0000 | 0.0101 ± 0.0091 | 0.0362 ± 0.0128 | 0.0032 ± 0.0034 | 0.0138 ± 0.0054 |
| Mean AMBER energy (kcal/mol) | -1763.26 ± 5.80 | -1660.94 ± 6.56 | -1633.60 ± 8.70 | -1994.46 ± 9.35 | -2190.30 ± 7.17 | -1728.63 ± 7.57 | -1606.74 ± 5.68 |
| Mean restraint violation (kcal/mol) | 3.08 ± 0.31 | 3.37 ± 0.30 | 1.66 ± 0.38 | 2.32 ± 0.43 | 4.49 ± 0.58 | 1.67 ± 0.83 | 2.33 ± 0.48 |
|  |  |  |  |  |  |  |  |
| Clash score validated by MolProbity | 0.27 ± 0.89 | 0.39 ± 0.79 | 0.46 ± 1.31 | 0.18 ± 0.55 | 0.09 ± 0.41 | 0.18 ± 0.55 | 0.09 ± 0.42 |
| Ramachandran plot^a^ (%) |  |  |  |  |  |  |  |
| in most favored regions | 92.3 | 95.8 | 83.5 | 82.7 | 85.6 | 87.2 | 93.3 |
| in additional allowed regions | 7.7 | 4.2 | 16.0 | 16.7 | 14.4 | 4.5 | 6.5 |
| in generously allowed regions | 0.0 | 0.0 | 0.0 | 0.6 | 0.0 | 0.0 | 0.2 |
| in disallowed regions | 0.0 | 0.0 | 0.4 | 0.0 | 0.0 | 3.8 | 0.0 |
| RMSD from the averaged coordinates^a^ (Å) |  |  |  |  |  |  |  |
| Backbone atoms | 0.21 ± 0.06 | 0.22 ± 0.06 | 0.68 ± 0.24 | 0.44 ± 0.18 | 0.29 ± 0.08 | 0.46 ± 0.14 | 0.41 ± 0.13 |
| Heavy atoms | 0.79 ± 0.16 | 0.66 ± 0.13 | 1.78 ± 0.41 | 1.47 ± 0.29 | 0.84 ± 0.18 | 1.34 ± 0.24 | 1.02 ± 0.18 |
| h: human.  ^a^ For residues K269-T294, K297-T325, F325-K349, K353-D378, L402-G427, L769-H793, and L797-T823 for ZF2, ZF3, ZF4, ZF5, ZF6, ZF10, and ZF11, respectively. | | | | | | | |

| **Supplemental Table 2. Summary of statistics of the final 20 structures of ZFAT ZFs** | | | | | | |
| --- | --- | --- | --- | --- | --- | --- |
| Domain Names | hZFAT ZF12 | hZFAT ZF13 | hZFAT ZF14 | hZFAT ZF15 | mZFAT ZF5 | mZFAT ZF8 |
| PDB IDs | 2RV0 | 2RV1 | 2RV2 | 2RV3 | 2RV4 | 2RV5 |
| NOE upper distance restraints |  |  |  |  |  |  |
| Intra-residual (|*i*-*j*| = 0) | 128 | 128 | 132 | 156 | 158 | 164 |
| Medium-range (1 ≤ |*i*-*j*| ≤ 4) | 209 | 190 | 304 | 239 | 187 | 254 |
| Long-range (|*i*-*j*| > 4) | 91 | 97 | 188 | 134 | 67 | 115 |
| Total | 428 | 415 | 624 | 529 | 412 | 533 |
| Dihedral angle restraints (ϕ and ψ) | 28 | 28 | 30 | 30 | 26 | 26 |
|  |  |  |  |  |  |  |
| CYANA target function value (Å^2^) | 0.0331 ± 0.0037 | 0.0087 ± 0.0011 | 0.0005 ± 0.0007 | 0.0079 ± 0.0004 | 0.0056 ± 0.0042 | 0.0072 ± 0.0044 |
| Mean AMBER energy (kcal/mol) | -1906.91 ± 11.01 | -1833.80 ± 5.91 | -1858.51 ± 7.21 | -1468.82 ± 6.47 | -2002.51 ± 8.75 | -1828.94 ± 6.39 |
| Mean restraint violation (kcal/mol) | 3.09 ± 3.90 | 2.50 ± 0.22 | 4.42 ± 0.39 | 2.27 ± 0.28 | 1.93 ± 0.53 | 4.00 ± 0.82 |
|  |  |  |  |  |  |  |
| Clash score validated by MolProbity | 0.19 ± 0.58 | 0.09 ± 0.41 | 2.30 ± 1.34 | 1.19 ± 1.19 | 0.26 ± 0.64 | 0.20 ± 0.61 |
| Ramachandran plot^a^ (%) |  |  |  |  |  |  |
| in most favored regions | 83.6 | 87.7 | 87.5 | 94.8 | 83.6 | 90.6 |
| in additional allowed regions | 16.0 | 10.8 | 12.5 | 5.0 | 16.0 | 9.4 |
| in generously allowed regions | 0.4 | 1.0 | 0.0 | 0.0 | 0.4 | 0.0 |
| in disallowed regions | 0.0 | 0.4 | 0.0 | 0.2 | 0.0 | 0.0 |
| RMSD from the averaged coordinates^a^ (Å) |  |  |  |  |  |  |
| Backbone atoms | 0.32 ± 0.08 | 0.82 ± 0.24 | 0.19 ± 0.05 | 0.34 ± 0.11 | 0.38 ± 0.10 | 0.20 ± 0.06 |
| Heavy atoms | 0.98 ± 0.18 | 1.71 ± 0.33 | 0.81 ± 0.15 | 0.89 ± 0.22 | 1.44 ± 0.26 | 0.88 ± 0.17 |
| h: human; m: mouse.  ^a^ For residues S829-E855, A879-G905, K907-H931, T933-T957, Q354-D378, and Y458-G482 for ZF12, ZF13, ZF14, ZF15, mouse ZF5, and mouse ZF8, respectively. | | | | | | |

| **Supplemental Table 3. Summary of statistics of the final 20 structures of ZFAT tandem ZFs** | | |
| --- | --- | --- |
| Domain Names | hZFAT ZF2-ZF3-ZF4 | hZFAT ZF3-ZF4-ZF5 |
| PDB IDs | 2RV6 | 2RV7 |
| NOE upper distance restraints |  |  |
| Intra-residual (|*i*-*j*| = 0) | 442 | 485 |
| Medium-range (1 ≤ |*i*-*j*| ≤ 4) | 779 | 1121 |
| Long-range (|*i*-*j*| > 4) | 405 | 514 |
| Total | 1626 | 2120 |
| Dihedral angle restraints (ϕ and ψ) | 84 | 82 |
|  |  |  |
| CYANA target function value (Å^2^) | 0.0956 ± 0.0258 | 0.7500 ± 0.1400 |
| Mean AMBER energy (kcal/mol) | -4486.73 ± 7.82 | -4627.00 ± 10.46 |
| Mean restraint violation (kcal/mol) | 6.06 ± 0.45 | 13.11 ± 0.96 |
|  |  |  |
| Clash score validated by MolProbity | 0.11 ± 0.26 | 0.18 ± 0.32 |
| Ramachandran plot^a^ (%) |  |  |
| in most favored regions | 89.2 | 88.0 |
| in additional allowed regions | 10.6 | 11.7 |
| in generously allowed regions | 0.0 | 0.2 |
| in disallowed regions | 0.2 | 0.1 |
| RMSD from the averaged coordinates^b^ (Å) |  |  |
| Backbone atoms | 0.16 ± 0.05, 0.31 ± 0.10, 0.24 ± 0.06 | 0.18 ± 0.06, 0.20 ± 0.06, 0.28 ± 0.09 |
| Heavy atoms | 0.79 ± 0.16, 0.87 ± 0.19, 1.01 ± 0.21 | 0.72 ± 0.19, 0.85 ± 0.20, 1.15 ± 0.24 |
| h: human.  ^a^ For residues K271-H350 and K297-D378 for ZF2-ZF3-ZF4 and ZF3-ZF4-ZF5, respectively.  ^b^ For residues F271-H293, Y299-H321, and F326-H349 for ZF2, ZF3, and ZF4 of ZF2-ZF3-ZF4; and Y299-H321, F326-H349, and Q354-H377 for ZF3, ZF4, and ZF5 of ZF3-ZF4-ZF5, respectively. | | |
